# A semi-quantitative assay of overall DNA methylation status using Methyl-CpG binding protein (MBD1)

**DOI:** 10.1186/1756-0500-5-234

**Published:** 2012-05-14

**Authors:** Chunxiao Zhang, Runsheng Li, Ruqian Zhao

**Affiliations:** 1 Key Laboratory of Animal Physiology & Biochemistry, Nanjing Agricultural University, Nanjing, 210095, People’s Republic of China

**Keywords:** Overall DNA methylation, MBD1, Slot blot assay

## Abstract

**Background:**

In mammals, DNA methylation at the 5-position of cytosine is the most essential epigenetic modification. Changes in the level of genome-wide DNA methylation (also known as overall DNA methylation) are associated with alterations in gene expression, thereby contributing to the phenotypic and physiological diversity. Current technologies for detecting overall DNA methylation either suffer from low sensitivity or require sophisticated equipment. Studies on domestic animals are hampered by the lack of complete and annotated genomic information.

**Results:**

Here we report a rapid slot blot method using methyl-CpG binding protein (MBD1) to exam the level of overall DNA methylation in pigs and chickens. Using this rapid approach, we determined the methylation status in various DNA samples of a Chinese indigenous (Erhualian) and a Western (Large White) breed of pigs. We also chose day 18 embryos (E18) and newly hatched chicks (D1) of a Chinese indigenous chicken breed (Wen’s yellow-feathered broiler chicken) for genome-wide DNA methylation analysis. The results revealed tissue- and breed-specific differences, as well as age-dependent variations, in the level of overall DNA methylation.

**Conclusion:**

The results showed that the slot blot assay is a sensitive, highly specific and convenient method for semi-quantitative estimation of overall DNA methylation with no species specificity. This method does not require sophisticated equipment, such as high performance liquid chromatography (HPLC), or expensive technologies like sequencing, thus providing a useful tool for overall DNA methylation studies on domestic animals.

## Background

DNA methylation of the CpG dinucleotide is one of the most crucial epigenetic modifications in mammals [1]. Its feature is a modification of the 5-position of the pyrimidine ring of cytosine that produces 5-methylcytosine (5mc). A large body of literature demonstrates that the level of genome-wide DNA methylation (also is referred to as overall DNA methylation) changes dynamically during the course of normal development, and this epigenetic regulation is critical in the determination of cell fate during embryogenesis [2,3]. For example, during the preimplantation stage, the mammalian genome becomes progressively demethylated, which is associated with the initiation of cellular differentiation of somatic cells [3].

On the contrary, impairment of methylation can damage assorted aspects of gene regulation, such as X chromosome inactivation [1], genomic imprinting [4] and retroviral silencing [5]. Additionally, environmental factors, such as ionizing radiation [6] and hormone exposure [7], could influence the overall level of DNA methylation, which results in altered molecular pathways and increased risk of diseases. Thus, DNA methylation may help explain the pathophysiology of diseases. Furthermore, the aberrant genome-wide DNA methylation also plays a critical role in tumorigenesis [8]. The overall DNA methylation level in tumor-derived genomic DNA is reported to be significantly reduced. Hypomethylation of overall DNA methylation in tumor tissue has become a common hallmark in a variety of malignancies, such as breast, colon, and blood cancers [9,10]. Recently, a large number of studies have focused on the methylation status of specific genes to identify the cancer associated changes in DNA methylation at specific loci. However, analysis of genome-wide DNA methylation could provide an overview that may be missed in studies limited to specific genes of interest.

In order to understand the role of overall DNA methylation in development and disease, a wide range of approaches have been developed [11,12]. Generally speaking, there are four groups of approaches for detecting the changes of DNA methylation at the genome-scale level. The first group is high-performance separation techniques, such as high performance liquid chromatography (HPLC), HPLC-mass spectrometry (HPLC-MS), high performance capillary electrophoresis (HPCE), etc. The separation techniques require expensive and sophisticated equipment and significant experimental experience to obtain reproducible results. In addition, the DNA samples have to be hydrolyzed. The second group is based on enzymatic/chemical approaches such as the methyl-acceptor assay and the chloroacetaldehyde assay. These methods are not as sensitive as the high-performance separation techniques, and sometimes their resolution is limited to endonuclease cleavage sites and incomplete DNA digestion. The third group is bisulfite sequencing, which relies on bisulfite treatment to deaminate the unmethylated cytosine into uracil, thus allowing differentiation of methylated (protected from deamination) and unmethylation cytosine by deep sequencing. Although this technique allows whole genome analysis, it is costly and requires significant bioinformatics expertise and well annotated genomic information of the species. The fourth group is mentioned in literature in which DNA samples are immobilized on membranes and the methylation level is detected by the anti-5mc antibodies. But in order to expose the epitopes, DNA must be denatured into single strand.

The methyl-CpG binding domain (MBD), on the contrary, can detect native double-stranded CpG-methylated DNA [13]. MBD is a family of mammalian proteins whose original function is to recruit transcriptional repression complexes to silence gene expression [14]. MBD1, MBD2, MBD4 and MeCP2 have high affinity to a symmetrically methylated CpG motif [15], and MBD1 binds more efficiently to densely methylated DNA [16,17] with sequence preference in the neighboring nucleotides [18]. Methods using the MBD family protein provide an advantage compared to the previously discussed ones because no pre-treatment or conversion of the sample DNA is required. Large numbers of detection protocols have focused on applying the methyl binding proteins to capture the methylated DNA, such as methylated-CpG island recovery assay (MIRA) [19], and MBD-isolated Genome Sequencing (MiGS) [17]. Because high-throughput DNA sequencing is employed, these approaches have extremely high sensitivity and can provide information about DNA methylation for specific DNA loci at a resolution of nearly 50–100 bp. However, high-throughput sequencing is expensive, time consuming and most importantly, depends on substantial species-specific genomic database, which is not always available for domestic animals.

Previous studies used MBD1 protein to determine the methylated lambda phage DNA (λDNA) with slot blot assay [20,21]. Here we extend the application of the assay for detecting the relative percentage of genomic DNA methylation by using the same MBD1 protein. The assay does not require high-performance separation equipment or methylation-sensitive restriction enzymes, yet provides a quick, simple and inexpensive semi-quantitative technique for the detection of methylated DNA. Using this rapid approach, we measured genome-wide DNA methylation levels in different DNA samples of a Chinese indigenous (Erhualian) and a Western (Large White) breed of pigs. Also, we chose day 18 embryo (E18) and newly hatched chicks (D1) of a Chinese indigenous chicken breed (Wen’s yellow-feathered broiler chicken) for this methylation level analysis. The semi-quantitative analysis revealed tissue- and breed-specific differences, as well as age-dependent variations in the overall DNA methylation level.

## Results

### Specificity of the slot blot assay

**Figure 1:**
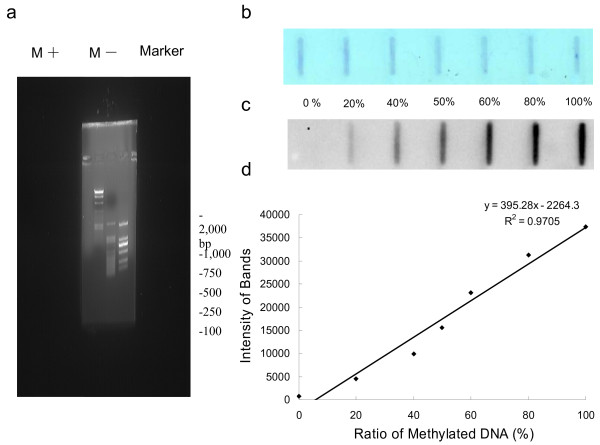
**Specificity of the slot blot assay using MBD1 protein. (a)** Methylation status of the λDNA assessed by *Hpa*II endonuclease restriction analysis shows that in vitro methylated λDNA (M+) was resistant to endonuclease cleavage, but unmethylated λDNA (M-) was not. **(b)** Methylated λDNA (100%) was added in increasing amounts to unmethylated λDNA (0%) to create a dilution series of methylated DNA from 0% to 100% in a total amount of 500 ng. The blot was stained with methylene blue to ensure the equal loading of total DNA. **(c)** The same blot detected by MBD1. **(d)** The observed band intensity was plotted against the methylation percentage. The regression equation with an adjusted R^2^ of 0.9705 was presented on figure.

As shown in Figure 1a, the specificity of the MBD1 protein was tested against *M.SssI*-methylated and non-methylated λDNA following the method described previously [20,21]. Serial dilution of methylated λDNA samples demonstrated increasing band intensity, indicating the status of DNA methylation (Figure 1c). The assay demonstrated high specificity, as signals were obtained only in the slots containing the methylated λDNA, which is consistent with previous publications [20,21]. A linear regression analysis showed quantitative recovery across the entire methylation range (0-100%), with a R^2^ of 0.9705 (Figure 1d). The blot stained with methylene blue demonstrated equal loading of total DNA on each slot (Figure 1b).

### Sensitivity of the slot blot assay

**Figure 2:**
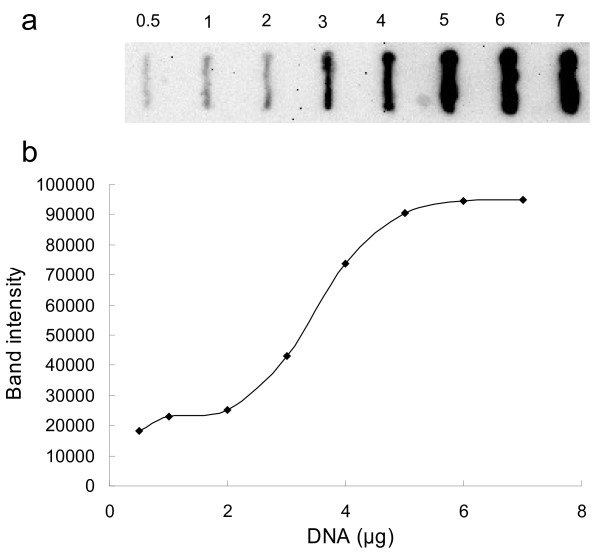
**Sensitivity of the slot blot assay using MBD1 protein. (a)** Increasing amount of genomic DNA ranging from 0.5 to 7 μg from leg muscle of D1 chicken was blotted onto the membrane. **(b)** The observed band intensity was plotted against the amount of DNA blotted.

We examined the sensitivity of methyl-CpG-binding by incubating recombinant MBD1 protein with membranes carrying different quantities of genomic DNA. Genomic DNA extracted from the muscle of D1 chicken was used, and the result suggested that the slot blot routinely allowed detection of genomic DNA to a sensitivity of 0.5 μg (Figure 2).

### Differences of overall DNA methylation levels among different tissues of two pig breeds

**Figure 3:**
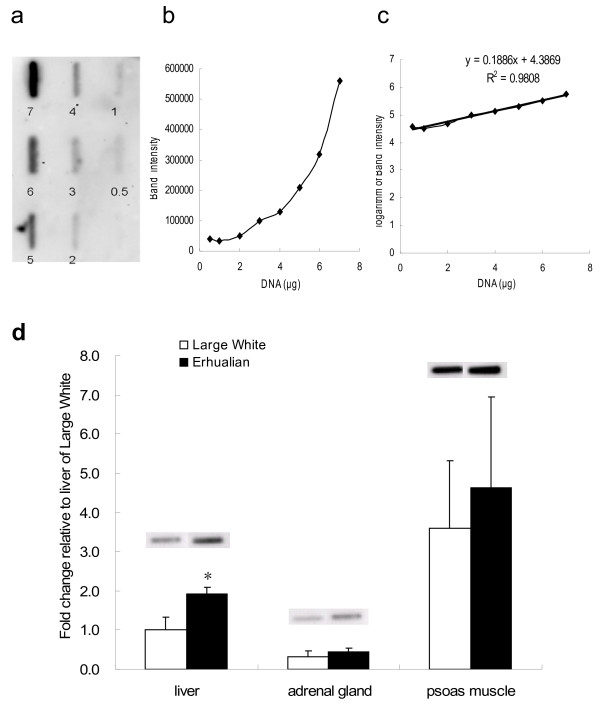
**Utilizing the slot blot assay to detect the overall DNA methylation level of newborn piglets. (a)** Increasing amount of porcine genomic DNA (a mix of all the samples detected) ranging from 0.5 to 7 μg (marked underneath each band) was blotted onto the membrane. **(b)** The observed band intensity was plotted against the amount of DNA blotted. **(c)** The logarithm of band intensity was plotted against the amount of DNA blotted. **(d)** 4 μg of genomic DNA from three types of tissues of male Large White and Erhualian piglets was used (n = 6). Data are presented as means ± SE. * means significant difference between pig breed (p < 0.05).

We utilized the slot blot assay to assess the level of overall DNA methylation among three types of tissues (liver, adrenal gland and psoas muscle) of two breeds of newborn piglets (n = 6). Increasing amounts of porcine genomic DNA (a mix of all the samples detected) were blotted onto the membrane (Figure 3a) to plot a standard curve with band intensity against DNA quantity (Figure 3b). The band intensity is log-linear to the DNA amount (Figure 3c). The result showed that the band intensity from 4 μg of DNA is within the linear range of the standard curve. We found that the most highly methylated DNA was from the psoas muscle. The differences between muscle and the other two tissues, liver and adrenal gland, were significant with p < 0.05 (Figure 3d). The degree of genomic DNA methylation is higher in Erhualian piglets than in Large White in all the three tissue types, and a significant breed difference was detected in liver (p = 0.034).

### Differences of overall DNA methylation levels among different tissues at two developmental stages in the chicken

**Figure 4:**
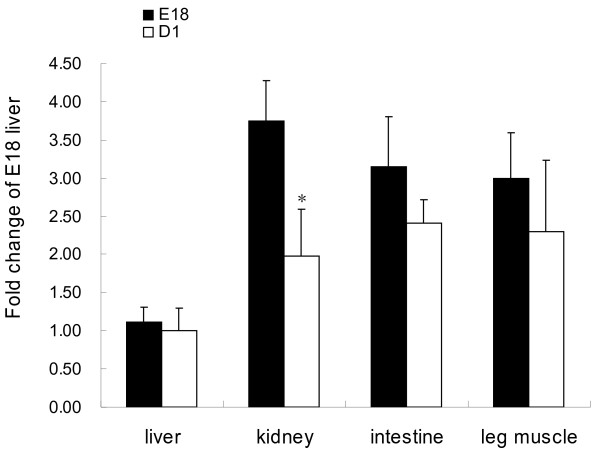
**Utilizing the slot blot assay to detect the overall DNA methylation level of chickens.** 3 μg of genomic DNA from 18 day embryo (E18) and new hatched (D1) chicken (n = 6) was used. Data are presented as means ± SE. Different superscripts indicate significant difference between bars (p < 0.05).

The overall DNA methylation levels of chicken liver, kidney, intestine, and leg muscle exhibited a decrease at D1 compared to E18 (n = 6). The age-related drop in overall DNA methylation level was significant in kidney (p = 0.05) (Figure 4). At E18, the overall DNA methylation level of kidney is significantly higher than that of liver. But at D1, the DNA methylation level did not exhibit significant difference among the tissues.

### Verification by using anti-5mc antibody

**Figure 5:**
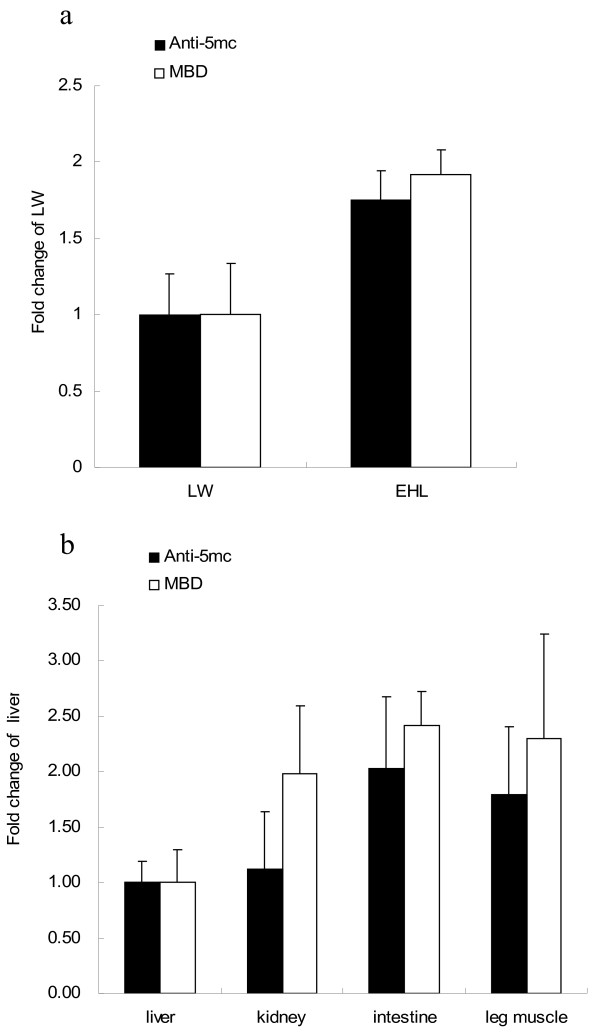
**Verification by using Anti-5mC antibody. (a)** The same genomic DNA from liver of male Large White and Erhualian piglets (n = 6) was detected by the anti-5mc antibody. **(b)** The identical genomic DNA of different tissues of chicken (D1) was detected by the anti-5mc antibody. The results are consistent with that detected by the MBD1 protein. The data are presented as means ± SE.

To confirm our slot blot assay, we compared two of the results with conventional Southern blotting with commercial anti-5mc antibody. We chose the DNA samples of the liver of two pig breeds, and D1 chicken tissues as examples. Genomic DNAs were denatured in a 95°C water bath and put on ice immediately. Then they were immobilized on nitrocellulose membranes and detected with commercial anti-5mc antibody (1:5000) followed by HRP-anti-mouse antibody. The pattern of overall DNA methylation in different tissues was consistent with that detected by our method (Figure 5a,b).

## Discussion

In this study, we demonstrated the feasibility of immobilizing genomic DNA on membranes with slot blotting followed by interaction with MBD1 protein for quantifying overall DNA methylation. Most of the currently available methods require DNA to be denatured by heating in order to fully expose the epitopes [22,23]. The assay developed in this study can avoid subjecting the specimen to rough denaturing conditions, as MBD1 could recognize 5mc in duplex DNA. The sensitivity of this assay is similar to other Southwestern immunoblotting assays with anti-5-methylcytosine antibody [24]. Although the sensitivity of the slot blot using MBD1 protein is relatively lower compared to methods such as HPLC or HPEC [11], it would meet the needs of those studies in which an semi-quantitative estimation is adequate and quantity of the DNA samples is not restricted.

Levels of methylated DNA are known to be tissue- and species-specific [6,25,26], and change during development [27]. Analysis of the overall DNA methylation level revealed that in normal human tissue, the most highly methylated DNA was from thymus and the least was from sperm [28]. Gama-Sosa [26] analyzed the overall DNA methylation extent in rats, mice and four types of monkeys, and they demonstrated that there were some similarities among tissues in mammals that they examined. For example, brain and thymus DNA were hypermethylated compared to most other organ DNA. Our result showed that in pig, muscle had the highest methylation level compared to the liver and adrenal gland. The physiological significance of these marked differences in overall DNA methylation level among the organs of a given species is not yet clear. Several lines of evidence demonstrate that high levels of DNA methylation are associated with gene silencing [1]. However, no apparent correlation is observed in the tissue-specific differences in levels of human overall DNA methylation and the extent of transcriptional activity [28]. Variations in the overall DNA methylation level of a certain type of tissue might indicate, to some extent, the net change of the transcriptional activity of all the genes in all the cell types and thus, could be related to differentiation and function of these tissues [26]. Our results also indicate that the overall DNA methylation level in the liver of swine differs between breeds; other tissues showed the same tendency, but no significant change was observed.

Developmental changes in DNA methylation will be helpful to understand the molecular basis of age-related physiological and pathological changes [29]. It has been illustrated that the overall DNA methylation levels decrease with age in human and mouse [30]. All the chicken tissues detected in the present study showed a decrease from E18 to D1. The reduction of the overall DNA level is a net effect of increases and decreases in methylation that occur across the whole genome [31]. The age-related decrease of methylation in CpG islands seems uncommon [32], whereas the age-related demethylation of repetitive sequence was common in human and other animals [33-35]. However, illustration of DNA methylation changes with age at specific loci is far from complete, and beyond the scope of our present study. In our study, the kidney showed a significant developmental change. Previous studies suggest that some organs, such as lung and kidney, underwent significant biochemical changes after birth [36-38]. Such biochemical changes are regulated by hormones [37] and may be related to the overall DNA methylation changes.

It is noted, however, that our assay is limited in offering the absolute values for the proportion of methylated cytosine, because fully methylated and unmethylated control DNA for swine and chicken are not available. However, an absolute percentage is not always necessary. For example, one research study aimed to analyze the relationship between an overgrowth phenotype of bovine fetuses and DNA methylation, and they compared the overall DNA methylation level of liver and placental cotyledon between the overgrowth groups and controls by using HPCE [39]. HPCE offered an absolute percentage of methylation, but actually a semi-quantitative analysis like our assay is adequate to serve the purpose.

The slot blot assay with MBD1 protein may also be useful for many different methylation detection applications that were not explored in this study. Its sensitivity, specificity and ease of use would allow for enhancing our understanding of the role of DNA methylation in different tissues of various species of animals under different situations.

## Methods

### Ethical statement

The slaughter and sampling procedures complied with the "Guidelines on Ethical Treatment of Experimental Animals" (2006) No. 398 set by the Ministry of Science and Technology, China and the Regulation regarding the Management and Treatment of Experimental Animals" (2008) No.45 set by the Jiangsu Provincial People's Government. The experiment was conducted following the guidelines of Animal Ethics Committee at Nanjing Agricultural University, China.

### DNA sample preparation

Genomic DNA was extracted from tissues by incubation in lysis buffer (20 mM Tris (pH 8), 20 mM EDTA, 2% SDS and 0.5 mg/ml Proteinase K) overnight at 55°C followed by phenol:chloroform extraction and ethanol precipitation. The extracted DNAs were quantified using the NanoDrop^TM^ 1000 Spectrophotometer (Thermo Scientific, USA). Each sample was diluted with distilled water into a final concentration of 100 ng/μl.

### Preparation of λDNA

We methylated 1 μg λDNA (Promega D1521) with 4U of the CpG methyltransferase *SssI* in 20 μl reaction volumes with 1 × NEBuffer2 and 160 μmol/l S-adenosylemthyionine for 2 h at 37°C (all reagents from New England Biolabs). To confirm the complete methylation, both the native double-stranded mock- and M.*SssI*-methylated λDNA were digested by *Hind*III, and then they were subjected to restriction analysis using the methylation sensitive enzyme *Hpa*II (all the enzymes from New England Biolabs) on 1% agarose gel.

Methylated λDNA (100%) was diluted with unmethylated λDNA (0%) to create a dilution series of 100%, 80%, 60%, 40%, 20%, 0% in a total amounts of 500 ng. We then used duplicate DNA dilutions to do the slot blot. One membrane was measured the methylation percentage as described, and the other was immersed in methylene blue stain for 5 min at room temperature and wash the membrane three times with water.

### Recombinant protein

Prof. Adrian P. Bird at the Welcome Trust Centre for Cell Biology University of Edinburgh, UK kindly donated the plasmid of 1 × MBD (pET-1 × MBD). We transformed the plasmids to BL21 (DE3). Two hundreds mls BL21 (DE3) cultures were induced by 1 mM IPTG and the recombinant His6-tagged MBD1 proteins were prepared by denaturation-renaturation procedure as previously described [20,21].

### Slot blot assay

The slot blot apparatus (Cleaver Model, 48 slots) was assembled using Whatman 3 MM filter and a pre-wetted nitrocellulose membrane, and it was connected to a vacuum pump. For slot blotting, 40 μl of DNA samples at the concentration of 100 ng/μl were taken and brought up to 200 ml with water, the unused slots were filled with 200 ml distilled water, and then drawn by vacuum and immobilized by heating at 80°C for one hour. The membrane was blocked for 2 h at room temperature in 5% skim milk powder in Tris–HCl buffered saline containing Tween-20 (0.025 M Tris–HCl, 0.15 M NaCl, pH 7.6, 0.05% Tween-20, TBST). The membrane was then incubated with TBST buffer containing 5% skim milk and 20 μg/ml purified recombinant MBD1 protein for 2 h at room temperature or, alternatively overnight at 4°C. The membrane was washed three times with TBST. The bound protein was detected by incubation with HisProbe-HRP (Thermo Scientific, USA) working solution (1:5000 of stock in 2.5% BSA TBST) for 1 h. Again, the membrane was washed three times with TBST and the blot was processed using the Supersignal West Dura Extended Duration Substrate (Thermo scientific, USA) according to the manufacturer’s introductions. Chemiluminescence was detected with Versa Doc^TM^ imaging system (BIO-RAD, USA) and intensity of each band on the slot was measured using the Quantity One Analysis Software (BIO-RAD, USA).

### Statistical analysis

All data are presented as the mean ± SEM. All statistical analyses were performed with two-way analysis of variance (ANOVA) using the general linear model (GLM) procedure of SPSS 17.0 for Windows, followed by post-hoc analysis using the S-N-K method. For single factor studies, comparison was performed with Student’s *T* test for independent samples. Differences were considered significant when p < 0.05.

## Conclusion

In conclusion, the slot blot assay is a sensitive, highly specific and inexpensive method for semi-quantitative estimation of overall DNA methylation. This assay does not require sophisticated equipments, or expensive technologies like sequencing, and also does not have the species specificity, thus providing a useful tool for the overall DNA methylation studies on domestic animals.

## Competing interests

All the authors declare that they have no competing interests.

## Authors’ contributions

RZ designed the study. CZ carried out the experiment, participated in the design of the study and drafted the manuscript. RL performed the statistical analysis and helped to draft the manuscript. All authors read and approved the final manuscript.

## References

[B1] Bird A DNA methylation patterns and epigenetic memory Genes Dev 2002 16 6 21 10.1101/gad.947102 11782440

[B2] Reik W Dean W Walter J Epigenetic reprogramming in mammalian development Science 2001 293 1089 1093 10.1126/science.1063443 11498579

[B3] Rougier N Bourc'his D Gomes DM Niveleau A Plachot M Paldi A Viegas-Pequignot E Chromosome methylation patterns during mammalian preimplantation development Genes and development 1998 12 2108 2113 10.1101/gad.12.14.2108 9679055 PMC317005

[B4] Li E Beard C Jaenisch R Role for DNA methylation in genomic imprinting Nature 1993 366 362 365 10.1038/366362a0 8247133

[B5] Walsh CP Chaillet JR Bestor TH Transcription of IAP endogenous retroviruses is constrained by cytosine methylation Nat Genet 1998 20 116 117 10.1038/2413 9771701

[B6] Pogribny I Raiche J Slovack M Kovalchuk O Dose-dependence, sex- and tissue-specificity, and persistence of radiation-induced genomic DNA methylation changes Biochem Biophys Res Commun 2004 320 1253 1261 10.1016/j.bbrc.2004.06.081 15249225

[B7] Xu N Azziz R Goodarzi MO Epigenetics in polycystic ovary syndrome: a pilot study of global DNA methylation Fertility and sterility 2010 94 781 783 e781 10.1016/j.fertnstert.2009.10.020 19939367 PMC2889203

[B8] Ehrlich M DNA methylation in cancer: too much, but also too little Oncogene 2002 21 5400 5413 10.1038/sj.onc.1205651 12154403

[B9] Robertson KD DNA methylation and human disease Nat Rev Genet 2005 6 597 610 16136652 10.1038/nrg1655

[B10] Terry MB Delgado-Cruzata L Vin-Raviv N Wu HC Santella RM DNA methylation in white blood cells: Association with risk factors in epidemiologic studies Epigenetics 2011 6 828 837 10.4161/epi.6.7.16500 21636973 PMC3154425

[B11] Fraga MF Esteller M DNA methylation: a profile of methods and applications BioTechniques 2002 33 636 649 632, 634 10.2144/02333rv01 12238773

[B12] Laird PW Principles and challenges of genomewide DNA methylation analysis Nat Rev Genet 2010 11 191 203 20125086 10.1038/nrg2732

[B13] Nan X Meehan RR Bird A Dissection of the methyl-CpG binding domain from the chromosomal protein MeCP2 Nucleic Acids Res 1993 21 4886 4892 10.1093/nar/21.21.4886 8177735 PMC311401

[B14] Veenstra GJC Bogdanovic O DNA methylation and methyl-CpG binding proteins: developmental requirements and function Chromosoma 2009 118 549 565 10.1007/s00412-009-0221-9 19506892 PMC2729420

[B15] Hendrich B Bird A Identification and characterization of a family of mammalian methyl-CpG binding proteins Mol Cell Biol 1998 18 6538 6547 9774669 10.1128/mcb.18.11.6538 PMC109239

[B16] Fujita N Shimotake N Ohki I Chiba T Saya H Shirakawa M Nakao M Mechanism of transcriptional regulation by methyl-CpG binding protein MBD1 Mol Cell Biol 2000 20 5107 5118 10.1128/MCB.20.14.5107-5118.2000 10866667 PMC85960

[B17] Nair SS Coolen MW Stirzaker C Song JZ Statham AL Strbenac D Robinson MW Clark SJ Comparison of methyl-DNA immunoprecipitation (MeDIP) and methyl-CpG binding domain (MBD) protein capture for genome-wide DNA methylation analysis reveal CpG sequence coverage bias Epigenetics 2011 6 34 44 10.4161/epi.6.1.13313 20818161

[B18] Clouaire T de Las Heras JI Merusi C Stancheva I Recruitment of MBD1 to target genes requires sequence-specific interaction of the MBD domain with methylated DNA Nucleic Acids Res 2010 38 4620 4634 10.1093/nar/gkq228 20378711 PMC2919722

[B19] Rauch TA Pfeifer GP DNA methylation profiling using the methylated-CpG island recovery assay (MIRA) Methods 2010 52 213 217 10.1016/j.ymeth.2010.03.004 20304072 PMC2910839

[B20] Jorgensen HF Adie K Chaubert P Bird AP Engineering a high-affinity methyl-CpG-binding protein Nucleic Acids Res 2006 34 e96 10.1093/nar/gkl527 16893950 PMC1540740

[B21] Cipriany BR Zhao R Murphy PJ Levy SL Tan CP Craighead HG Soloway PD Single molecule epigenetic analysis in a nanofluidic channel Anal Chem 2010 82 2480 2487 10.1021/ac9028642 20184350 PMC2839087

[B22] Li M Hu SL Shen ZJ He XD Tao SN Dong L Zhu YY High-performance capillary electrophoretic method for the quantification of global DNA methylation: application to methotrexate-resistant cells Anal Biochem 2009 387 71 75 10.1016/j.ab.2008.12.033 19167340

[B23] Stach D Schmitz OJ Stilgenbauer S Benner A Dohner H Wiessler M Lyko F Capillary electrophoretic analysis of genomic DNA methylation levels Nucleic Acids Res 2003 31 E2 10.1093/nar/gng002 12527791 PMC140527

[B24] Oakeley EJ Podesta A Jost JP Developmental changes in DNA methylation of the two tobacco pollen nuclei during maturation Proc Natl Acad Sci USA 1997 94 11721 11725 10.1073/pnas.94.21.11721 9326677 PMC23612

[B25] Gonzalgo ML Jones PA Mutagenic and epigenetic effects of DNA methylation Mutat Res 1997 386 107 118 10.1016/S1383-5742(96)00047-6 9113112

[B26] Gama-Sosa MA Midgett RM Slagel VA Githens S Kuo KC Gehrke CW Ehrlich M Tissue-specific differences in DNA methylation in various mammals Biochim Biophys Acta 1983 740 212 219 10.1016/0167-4781(83)90079-9 6860672

[B27] Thompson RF Atzmon G Gheorghe C Liang HQ Lowes C Greally JM Barzilai N Tissue-specific dysregulation of DNA methylation in aging Aging Cell 2010 9 506 518 10.1111/j.1474-9726.2010.00577.x 20497131 PMC2935175

[B28] Ehrlich M Gama-Sosa MA Huang LH Midgett RM Kuo KC McCune RA Gehrke C Amount and distribution of 5-methylcytosine in human DNA from different types of tissues of cells Nucleic Acids Res 1982 10 2709 2721 10.1093/nar/10.8.2709 7079182 PMC320645

[B29] Petronis A Human morbid genetics revisited: relevance of epigenetics Trends in genetics : TIG 2001 17 142 146 10.1016/S0168-9525(00)02213-7 11226607

[B30] Fuke C Shimabukuro M Petronis A Sugimoto J Oda T Miura K Miyazaki T Ogura C Okazaki Y Jinno Y Age related changes in 5-methylcytosine content in human peripheral leukocytes and placentas: an HPLC-based study Ann Hum Genet 2004 68 196 204 10.1046/j.1529-8817.2004.00081.x 15180700

[B31] Dunn BK Hypomethylation: one side of a larger picture Ann N Y Acad Sci 2003 983 28 42 10.1111/j.1749-6632.2003.tb05960.x 12724210

[B32] Tra J Kondo T Lu Q Kuick R Hanash S Richardson B Infrequent occurrence of age-dependent changes in CpG island methylation as detected by restriction landmark genome scanning Mech Ageing Dev 2002 123 1487 1503 10.1016/S0047-6374(02)00080-5 12425956

[B33] Hornsby PJ Yang L Gunter LE Demethylation of satellite I DNA during senescence of bovine adrenocortical cells in culture Mutat Res 1992 275 13 19 10.1016/0921-8734(92)90004-9 1372683

[B34] Suzuki T Fujii M Ayusawa D Demethylation of classical satellite 2 and 3 DNA with chromosomal instability in senescent human fibroblasts Exp Gerontol 2002 37 1005 1014 10.1016/S0531-5565(02)00061-X 12213551

[B35] Howlett D Dalrymple S Mays-Hoopes LL Age-related demethylation of mouse satellite DNA is easily detectable by HPLC but not by restriction endonucleases Mutat Res 1989 219 101 106 10.1016/0921-8734(89)90020-9 2538731

[B36] Powell JT Whitney PL Postnatal development of rat lung. Changes in lung lectin, elastin, acetylcholinesterase and other enzymes The Biochemical journal 1980 188 1 8 7406872 10.1042/bj1880001 PMC1162529

[B37] Imamura Y Iwamoto K Yanachi Y Higuchi T Otagiri M Postnatal development, sex-related difference and hormonal regulation of acetohexamide reductase activities in rat liver and kidney J Pharmacol Exp Ther 1993 264 166 171 8423525

[B38] Neiss WF Klehn KL The postnatal development of the rat kidney, with special reference to the chemodifferentiation of the proximal tubule Histochemistry 1981 73 251 268 10.1007/BF00493025 7327946

[B39] Hiendleder S Mund C Reichenbach HD Wenigerkind H Brem G Zakhartchenko V Lyko F Wolf E Tissue-specific elevated genomic cytosine methylation levels are associated with an overgrowth phenotype of bovine fetuses derived by in vitro techniques Biol Reprod 2004 71 217 223 10.1095/biolreprod.103.026062 15028629

